# Outcomes of Radioactive Iodine (^131^I) Therapy among Hyperthyroid patients

**DOI:** 10.12669/pjms.39.6.7567

**Published:** 2023

**Authors:** Muhammad Sajjad Ali Khan, Arshad Hussain, Shahzad Ahmad, Muhammad Harris Shah

**Affiliations:** 1 Muhammad Sajjad Ali Khan, FCPS Medicine. North West Teaching Hospital & Research Center, Peshawar, Pakistan; 2 Arshad Hussain, MRCP. North West Teaching Hospital & Research Center, Peshawar, Pakistan; 3 Shahzad Ahmad, FCPS Medicine. North West Teaching Hospital & Research Center, Peshawar, Pakistan; 4 Muhammad Harris Shah, MRCP. North West Teaching Hospital & Research Center, Peshawar, Pakistan

**Keywords:** I-131, Radioactive Iodine, Hyperthyroidism, Treatment Effectiveness

## Abstract

**Objective:**

The present study aimed to assess the thyroid outcomes six-months after radioactive Iodine-131 therapy (RIT) among hyperthyroid patients and identify the factors associated with them.

**Methods:**

This retrospective observational study was conducted at the department of Nuclear Medicine and Molecular Imaging, Northwest General Hospital & Research Centre during 2013 to 2019. For the study purpose, the thyroid outcomes of 153 hyperthyroid patients were studied retrospectively for six months after RIT. The data was obtained from the medical records. Patient baseline characteristics, clinical features, laboratory investigations, results of thyroid imaging, and therapeutic investigations were acquired and recorded in a structured questionnaire.

**Results:**

Out of the 153 screened records of hyperthyroid patients, 19.6% became euthyroid, 9.2% remained hyperthyroid, and 25.5% developed hypothyroidism after six months of RIT. The observed remission rate by the end of six months was 80.95%. Three months post-RIT, gender and RAI doses had a significant effect on thyroid function. The frequency of hypothyroidism was higher among those treated with an RAI dose of ≤ 20 mCi (83.0%) than those treated with a higher dose > 20 mCi (17%). Moreover, most patients receiving > 20 mCi radioiodine became euthyroid (64.5%). Similar outcomes were observed after six months of the therapy, except gender was replaced by etiologies of hyperthyroidism (p=0.009).

**Conclusion:**

Radioactive iodine (131-I) therapy is effective for the treatment of hyperthyroidism. However, the appropriate dose is still debatable, as there was a high incidence of hypothyroidism post-therapy.

## INTRODUCTION

Endocrine diseases are increasing rapidly worldwide, specifically in Asia.^1^ The pathophysiology of thyroid disorders is mainly linked to alterations in the levels of Thyroid Stimulating Hormone (TSH), triiodothyronine (T3), and thyroxine (T4). Hyperthyroidism is characterized by a low TSH while high T3 and T4 levels.^2^ The incidence of hyperthyroidism in Pakistan is lower than hypothyroidism, i.e., 2%.^3^ Furthermore, Graves’s disease contributes to 90% of the hyperthyroid cases, following toxic multinodular goiter mostly diagnosed among adult patients. In addition, five percent of patients are caused by toxic thyroid adenoma resulting from excessive thyroid hormone release^4^, while a few cases are also the result of sub-acute thyroiditis.^5^

Radioactive iodine ^131^I (RAI) therapy has been used as a first or second-line treatment approach for hyperthyroidism over the past few decades. The therapy intends to disrupt the activity of thyroid tissues to control hyperthyroidism and reach the euthyroid or hypothyroid stage. Although, it is a safe and effective definitive treatment for hyperthyroidism, with 100% cure after one or more treatments.^6^ There has been persistent debate on the radioactive iodine 131I (RAI) dose selection and the factors associated with the treatment success.^7^ There are several proposed regimens for ^131^I administration, i.e., low doses, fixed doses 185, 370, 555 MBq (5-15.0 mCi), and other calculated doses based on the size of the thyroid gland uptake of Radioactive Iodine and outcomes of RAI.

The dose calculation of Radioactive Iodine is necessary for therapeutic optimization considering the need to expose the body to the lowest possible radiation dose.^8^ Despite years of research and experimentation, there is still a lack of agreement on the appropriate dose regimen.^7^ Few studies suggest that a satisfactory remission rate is achieved while administering quite low doses of ^131^I determined using the dosimetric protocol, which is complex and expensive. However, adopting a fixed-dose method is rather a simplified approach with cost-saving advantages^9^. The cure rate associated with fixed doses and advantages of calculated dose over a fixed-dose regimen cannot be fully demonstrated.^9^

Among the factors associated with the low success rate of RIT are male gender, the severity of hyperthyroidism, enlargement of thyroid gland, and marked goitre^10^, but little is known about it in our country. We aimed to assess the thyroid outcomes after six months of radioactive Iodine-131 therapy (RIT) among hyperthyroid patients and identify the associated factors.

## METHODS

A retrospective observational study was conducted at the department of Nuclear Medicine and Molecular Imaging, Northwest General hospital & Research Centre during 2013 to 2019. The clinical records of hyperthyroid patients (Graves Disease, Multinodular Goiter, and hot nodules) treated with^131^I therapy were studied retrospectively. Pregnant or lactating female patients and those with a history of thyroidectomy were excluded from the study.

### Ethical Approval

It was obtained from the ethical review board of Northwest General Hospital & Research Center [Reference No; NwGH/EC/05; Dated 11-05-2020].

Thyrotoxicosis was determined based on suppressed TSH (< 0.01 mIU/L), elevated free T4 (FT4), and/or total T3 (TT3) values. While TSH levels between (0.3 to < 0.01 mIU/L) were observed on more than one occasion over several months and normal FT4 and TT3 values suggested Subclinical thyrotoxicosis. The etiology of hyperthyroidism was established by clinical features, laboratory investigations, and results of thyroid imaging.

Patients were examined three and six months after radioiodine administration. Thyroid profile was studied to determine the thyroid function (hyperthyroidism, hypothyroidism, euthyroidism), and post-RIT outcomes (remission and failure). Remission was labelled if the clinical and laboratory evidence suggested stable euthyroidism or hypothyroidism after six months of the radioactive iodine (^131^I) therapy. If the hypothyroidism persisted or relapsed, the therapy was considered failed.

### Statistical analysis

It was performed using SPSS 25.0; continuous variables were presented as mean ± SD and categorical variables as frequencies and percentages. The Chi-square test was used to compare the categorical variables, and a p-value less than 0.05 was considered statistically significant.

## RESULTS

A total of 153 patients (103 females and 50 males) with a mean age of 48.18 ± 12.60 years were included. 61.4% of patients received the 1^st^ dose of RAI ≤ 20 mCi, while 38.6% received a dose > 20 mCi. Furthermore, 5.2% were given a second dose > 20 mCi (Table-I).

**Table-I:** Baseline and clinical characteristics of all patients (n=153).

Variables		N(%)
Age (years), mean±SD		48.18±12.60
Sex	Female	103(67.3)
	Male	50(32.7)
Comorbidity	Yes	62(40.5)
	No	91(59.5)
Number of co-morbidities	One	26(17.0)
	Two	24(15.7)
	three	7(4.6)
	More than 3	5(3.3)
1^st^ Dose of RAI	≤ 20 mCi	94(61.4)
	> 20 mCi	59(38.6)
2^nd^ Dose of RAI	> 20 mCi	8(5.2)
	Nil	145(94.77)
Diagnosis	Graves’ disease	79(51.63)
	MNG	10(6.53)
	Normal functioning thyroid gland	2(1.30)
	Others	62(40.52)

The thyroid status was observed after three and six months post-RIT; 19.6% became euthyroid, 25.5% became hypothyroid, and 9.2% remained hyperthyroid after six months of RIT (Table-II). After therapy, the overall remission rate was 80.95%. No statistically significant association was found between Post-RIT outcome (remission and failure) and baseline characteristics, including age, gender, nationality, etiology of hyperthyroidism, co-morbidities, Pre RAI-TSH, and dose of RAI.

**Table-II:** Thyroidal status among the study population at three and six months.

Thyroidal status		N (%)
Three months	Euthyroidism	31(20.3)
	Hyperthyroidism	53(34.6)
	Hypothyroidism	47(30.7)
	NA	22(14.4)
Six months	Euthyroidism	30(19.6)
	Hyperthyroidism	14(9.2)
	Hypothyroidism	39(25.5)
	NA	70(45.8)

NA-Not Applicable.

When compared on the basis of post-RIT thyroid function (hyperthyroidism, euthyroidism, and hypothyroidism), a significant difference was observed in the thyroidal status with respect to gender (p=0.015). Moreover, 1^st^ and 2^nd^ RAI dose also significantly affected the thyroid function (p=0.007). It can be seen that the frequency of hypothyroidism was higher among those treated with an RAI dose of ≤ 20 mCi (83.0%) than those treated with a higher dose > 20 mCi (17%) (Table-III).

**Table-III:** Post-RIT thyroid function after three months with respect to patient baseline characteristics.

Variables		Post-RIT thyroid function (3 months)				p-value

		Euthyroidism (n=31)	Hyperthyroidism (n=53)	Hypothyroidism (n=47)	NA (n=22)	
Gender	Female	23(74.2)	35(66.0)	25(53.2)	20(90.9)	0.015*
	Male	8(25.8)	18(34.0)	22(46.8)	2(9.1)	
No. of co-morbidities	One	5(31.3)	10(41.7)	8(57.1)	3(37.5)	0.584
	Two	9(56.3)	8(33.3)	3(21.4)	4(50.0)	
	three	2(12.50)	3(12.5)	1(7.1)	1(12.5)	
	More than 3	-	3(12.5)	2(14.3)	-	
Diagnosis	Graves’ disease	10(32.3)	30(56.6)	26(55.3)	13(59.1)	0.141
	MNG	2(6.5)	4(7.5)	5(10.6)	-	
	Normal functioning thyroid gland	2(6.5)	-	-	-	
	Others	17(54.8)	19(35.8)	16(34.0)	9(40.9)	
1^st^ Dose of RAI	≤20 mCi	11(35.5)	34(64.2)	39(83.0)	10(45.5)	0.000*
	>20 mCi	20(64.5)	19(35.8)	8(17.0)	12(54.5)	
2^nd^ Dose of RAI	>20 mCi	-	7(13.2)	1(2.1)	-	0.027*
	Nil	31(100)	46(86.7)	46(97.87)	22(100)	

* p<0.05 is considered statistically significant.

There was a significant association between diagnosis and post-RIT thyroid function; a higher proportion of GD was observed among euthyroid, hypo, and hyperthyroid patients (p=0.009). Similarly, RAI dose also significantly affected thyroid function (p<0.01) (Table-IV).

**Table-IV:** Post-RIT thyroid function after six months with respect to patient baseline characteristics.

Variables		Post-RIT thyroid function (6 months)				p-value

		Euthyroidism (n=30)	Hyperthyroidism (n=14)	Hypothyroidism (n=39)	NA (n=70)	
Gender	Female	24(80.0)	9(64.3)	26(66.7)	44(62.9)	0.409
	Male	6(20.0)	5(35.7)	13(33.3)	26(37.1)	
Number of co-morbidities	One	6(31.6)	2(40.0)	1(20.0)	17(51.5)	0.752
	Two	8(42.1)	2(40.0)	3(60.0)	11(33.3)	
	three	2(10.5)	1(20.0)	1(20.0)	3(9.1)	
	More than 3	3(15.8)	-	-	2(6.1)	
Diagnosis	Graves’ disease	17(56.7)	10(71.42)	27(69.2)	25(35.7)	0.009*
	MNG	1(3.3)	1(7.1)	1(2.6)	8(11.4)	
	Normal functioning thyroid gland	1(3.3)	-	-	1(1.4)	
	Others	11(36.7)	3(21.4)	11(28.2)	36(51.4)	
1^st^ Dose of RAI	≤20 mCi	11(36.7)	9(64.3)	33(84.6)	41(58.6)	0.001*
	>20 mCi	19(63.3)	5(35.7)	6(15.4)	29(41.4)	
2^nd^ Dose of RAI	>20 mCi	4(13.3)	2(14.3)	2(5.1)	-	0.004*
	Nil	26(86.6)	12(85.7)	37(94.8)	70(100)	

* p<0.05 is considered statistically significant.

## DISCUSSION

Endocrinologists usually prefer a calculated dose of ^131^I therapy to treat Graves’ hyperthyroidism. Although the higher doses are associated with high RAI success rate but the unnecessary absorption of radiation dose must be keenly regulated as per the As Low As Reasonably Achievable (ALARA) principle.^11^ While on the other hand, the treatment failure also exposes the patient to additional ^131^I therapy. The present study results showed that the overall remission rate was 80.95%, similarly reported in a local study.^12^ Surprisingly, Wong et al. reported a much higher radioactive iodine treatment success rate, i.e., 93.3%. A randomized controlled trial studying the comparative success rate of fixed and calculated ^131^I dose suggested a success rate of 71% vs. 58% with fixed-dose and calculated dose therapy, respectively.^13^ Whereas a meta-analysis established inconclusive outcomes in relation to fixed dose and calculated dose.^14^ Furthermore, Traino and colleagues calculated the effect of three different ionizing radiation doses with 400 Gray on the thyroid gland; they reported the highest cure rate of 97%.^15^ There has been a significant difference in the success rate with low and high-dose RAI therapy.^16^,^17^

Assessing the thyroid function post RIT, we found that 19.6% were euthyroid, 25.5% were hypothyroid, while 9.2% remained hyperthyroid following the treatment protocol for six months. Likewise, a study including 84 patients followed for four to five months after RAI therapy demonstrated 16.6% euthyroid and 58.3% hypothyroid cases.^18^ Another similar study from Malaysia reported that 32.9% of the hyperthyroid patients developed hypothyroidism, and 19.7% became euthyroid within one-year post RAI therapy while 47.4% remained hyperthyroid.^19^ In an Indian study of 158 patients, 74.2% of the enrolled hyperthyroid patients developed hypothyroidism, 22.6% became euthyroid, and 1.2% remained thyrotoxic one year after RAI therapy.^20^

About the incidence of hypothyroidism post-RAI therapy, Shinto et al. specified that a higher proportion of hypothyroidism is associated with higher RAI doses (>10 mCi).^20^ Also supported by a study conducted by El-Sayed, it was reported that 48.3% of the patients receiving 15 mCi dose developed hypothyroidism at six months post RAI.^21^ The incidence of hypothyroidism (after six months) in the present study was greater among the patients receiving ≤ 20 mCi dose of RAI (84.6%) than those receiving a much higher dose (> 20 mCi) of radioiodine (15.4%).

Consistent with other studies^11^,^22^, we found no significant effect of age, gender, nationality, etiology of hyperthyroidism, co-morbidities, Pre RAI-TSH, and RAI doses on remission or failure of RIT. Whereas a significant effect of gender and RAI dose was observed on the thyroid function (incidence of hypothyroidism/euthyroidism) after three months of the treatment. There was no significant gender-based difference after six months, while etiologies of hyperthyroidism and RAI doses significantly affected thyroid function by this follow-up. There are both supporting and contrasting findings with respect to the factors affecting thyroid function after RIT; Mohamed et al. found a significant effect of gender and antithyroid drug use on the outcomes of post RAI and thyroid function^19^. Ghadban WK et al. reported no significant association between gender and thyroid functioning^23^, which is also supported by Allahabadia et al.^24^

Radioactive iodine therapy is generally considered a safe and effective treatment for hyperthyroidism but there is still a need to better understand the real-world outcomes and experiences of patients who receive this treatment. This observational study can help identify potential areas for improvement in the delivery of care and help identify patient factors that may influence treatment outcomes. The study contributes to the development of evidence-based guidelines for the treatment of hyperthyroidism. This can help ensure that patients receive the most appropriate and effective treatments for their condition. Overall, a research study on the outcomes of radioactive iodine therapy among hyperthyroid patients can have significant implications for clinical practice, patient care, and the development of evidence-based treatment guidelines.

### Limitations

It includes the retrospective nature of the study. It also lacks to report adverse effects with respect to the administration of ^131^I as the analysis was based on the limited data of the medical records. Furthermore, the impact of thyroid-stimulating immunoglobulin assays (TSIS) on treatment outcomes wasn’t estimated.

## CONCLUSION

In conclusion, Radioactive Iodine-131 therapy is highly effective for the treatment of hyperthyroidism. However, a high incidence of hypothyroidism was observed after the therapy. Among the factors associated with post RIT thyroid function, gender and RAI dose showed significant association.

### Authors’ Contribution:

SA is responsible for the concept and study design.

MSAK contributed to the data collection and literature review.

**MHS** are responsible for data analysis and interpretation and drafting of the manuscript.

**AH** contributed to the critical review, revision and final approval of the study.

All the authors are equally responsible and accountable for the accuracy and integrity of the work.
